# Results of robotic TAPP and conventional laparoscopic TAPP in an outpatient setting: a cohort study in Switzerland

**DOI:** 10.1007/s00423-022-02552-2

**Published:** 2022-05-24

**Authors:** Stephan Gerdes, Reint Burger, Georg Liesch, Barbara Freitag, Michele Serra, René Vonlanthen, Marco Bueter, Andreas Thalheimer

**Affiliations:** 1 Department of Visceral and Transplantation Surgery, University Hospital of Zürich, Rämistrasse 100, CH-8091 Zürich, Switzerland; 2 Department of Surgery, Spital Männedorf, Asylstrasse 10, CH-8708 Männedorf, Switzerland

**Keywords:** Inguinal hernia, Laparoendoscopic inguinal hernia surgery, Robotic-assisted, Outpatient

## Abstract

**Purpose:**

Recently, robotic surgery has been increasingly performed in hernia surgery. Although feasibility and safety of robot-assisted inguinal hernia repair in an inpatient setting have been already shown, its role in outpatient hernia surgery has not yet been investigated. Thus, this study aimed to compare robot-assisted TAPP (r-TAPP) and conventional laparoscopic TAPP (l-TAPP) in an outpatient setting.

**Methods:**

A prospective database of patients with inguinal hernia treated by l-TAPP or r-TAPP in an outpatient setting during a 1-year period was analyzed in a comparative cohort study. All patients underwent a check-up appointment with their surgeon within 3 days and 6 weeks postoperatively. Data on surgical time, perioperative complications, and postoperative pain were collected. Pain was recorded by using a Verbal Rating Scale (VRS).

**Results:**

Overall, outpatient laparoendoscopic inguinal hernia repair was performed in 58 patients (29 l-TAPP; 29 r-TAPP). Mean age was 57 years (21–81), mean BMI 24.5 kg/m2 (19–33) with no differences between both groups. Most patients reported none or only a low postoperative pain level in both groups (89.6% in l-TAPP group; 100% in r-TAPP), while there was a trend for less pain after r-TAPP. In both groups, there was one case of postoperative hematoma, which was successfully treated by conservative means. No other complications occurred during follow-up in either group and there was no 30-day-readmission, no unplanned overstay or any 30-day mortality in the cohort.

**Conclusion:**

Robot-assisted inguinal hernia surgery can be safely performed in an outpatient setting with a tendency to less pain when compared to the conventional laparoscopic technique. Cost-effectiveness and cost-coverage of outpatient robot-assisted inguinal hernia surgery must be further investigated in times of limited health cost resources and diagnosis-related medical reimbursements.

## Introduction

Surgical treatment of inguinal hernia is based on well-established international guidelines [1, 2]. Due to shorter hospital stay and lower postoperative pain, laparoendoscopic techniques are currently considered to be the standard inpatient treatment for inguinal hernia [2]. Due to cost constraints and patient requests, hernia operations are being increasingly performed in outpatient settings, with laparoendoscopic technique being repeatedly demonstrated to be safe and feasible [3, 4].

In recent years, robotic surgery has attracted enormous attention and the number of robot-assisted operations in general and visceral surgery has risen. Advantages for the surgeon include improved ergonomics, a better visualization, and a higher degree of freedom in the angulation of instruments as well as elimination of any tremor [5]. As a consequence, robotic technology has become increasingly more attractive for hernia surgery as well. The relatively high costs associated with robotic technology still limit its widespread use. While sufficient cost coverage seems basically possible in an outpatient setting, there is limited data on the safety and feasibility of outpatient robotic inguinal hernia surgery.

In this comparative cohort study, we report our experience with patients who underwent robotic TAPP (r-TAPP, transabdominal pre-peritoneal repair) in an outpatient setting and compare the short-term outcome to patients who underwent outpatient laparoscopic TAPP (l-TAPP).

## Methods

Data of all consecutive outpatient inguinal hernia patients in a single institution in Switzerland were prospectively collected between 01/-2020 and 12/-2020. No patient was excluded. Data was analyzed using descriptive statistics using Excel (Microsoft Corporation) and SPSS version 26.0 (IBM Inc., Chicago, USA). All included patients gave consent to use their data for research purposes via a general consent.

### Patients

All outpatient inguinal hernia patients were treated either by l-TAPP or r-TAPP (da Vinci Xi, Intuitive Surgical, Inc., Sunnyvale, CA). Inclusion criteria were based on standards of the health department of the canton of Zurich regulating the reimbursement for in- and outpatient treatment. We therefore included all consecutive patients who were eligible for outpatient surgery between 01/2020 and 12/2020. We also included patients that specifically opted for an outpatient setting and refused to be hospitalized, even if they would have formally qualified for a hospitalization. All other patients were treated as inpatients and were thus not included in our analysis. Further, the decision whether to use r-TAPP or l-TAPP did not follow a scientific randomization or an unintended selection process of the authors, but was rather due to availability of the robotic system as well as to patients’ request.

Patient characteristics (age, BMI, ASA classification, and existing anticoagulation), operative time (incision to suture) and postoperative pain were recorded. All patients received a standardized prescription for NSAIDs postoperatively with the corresponding dosage recommendation. Pain was assessed using the Verbal Rating Scale (VRS) with a four-point list (no pain, mild, moderate or severe pain). Postoperative pain was recorded directly after the operation in the recovery room and shortly before patients were discharged from the hospital.

### Operative technique

Patients were placed in a supine position. The operative technique was similar in both groups. For the r-TAPP, the robotic system was positioned on the patients right. Trocar positioning was standardized with two 5 mm and one 8 mm trocars on a horizontal line at the umbilical level in case of l-TAPP (5 mm videoscope) and three 8 mm DaVinci trocars 18 cm above the symphysis on a horizontal line with 8 cm distance from each other in case of r-TAPP. All surgeons performing r-TAPP (RB, GL, and AT) were certified Da Vinci surgeons. The docking procedure of the DaVinci Xi-system was performed following the company’s recommendations. Both procedures were performed in a Trendelenburg position. The peritoneum was incised, the hernia reduced. In case of a medial hernia, the transverse fascia was regularly sutured in EHS (European hernia society) M3 hernia using a V-Lock absorbable. The inguinal and femoral gap were covered with an inserted mesh (BARD 3D Lightmesh 10 × 15 cm) without further fixation. The peritoneum was closed with a running suture using V-Lock 3–0 absorbable. In case of a bilateral inguinal hernia both sides were fixed in the same operation.

## Results

### Patient characteristics

A total of 58 outpatient patients with a primary inguinal hernia, corresponding to 32.9% of all patients (in- and outpatient, *n* = 176) treated with inguinal hernia surgery in our department during the study period, were analyzed: 29 patients were treated with l-TAPP and 29 patients were treated with r-TAPP. No patient received open day-care surgery during this observation period. The decision whether to use a laparoscopic or robotic technique depended on the availability of the robotic system and thus also reflects the situation in a non-academic regional hospital. In each case, surgery was performed by an experienced consultant surgeon. In the group of l-TAPP, 83% of patients were male, 17% female. The average age was 53 years old (range 21–82) with an average BMI of 25 kg/m^2^ (range 19–33). In the group of r-TAPP, 93% of patients were male, 7% female. The average age in the robotic group was 62-years old (range 36–81) with an average BMI of 24 kg/m^2^ (range 20–29). The mean ASA classification in both groups was 2.

In the group of l-TAPP, we found 23 unilateral and 4 bilateral inguinal hernia, 1 unilateral femoral, and one bilateral femoral hernia. In the r-TAPP group, 26 unilateral and 3 bilateral inguinal hernia were diagnosed. An indirect inguinal hernia was most often found in the l-TAPP group (62%) with 68% in the r-TAPP group, respectively (Table 1).

**Table 1:** Patient characteristics

		l-TAPP bilateral (*n* = 5)		l-TAPP unilateral (*n* = 24)		r-TAPP bilateral (*n* = 3)		r-TAPP unilateral (*n* = 26)	
		Mean		Mean		Mean		Mean	
Age (years)		55.0		52.8		54.0		62.7	
Gender	M		4		20		3		24
	F		1		4		0		2
BMI (kg/m^2^)		23		25		25		24	
ASA		2		2		2		2	
Anticoagulation	Yes		0		2		0		2
	No		5		22		3		24
Classification (EHS)	M1				1				
	M2		1		2				3
	M3		1		2				
	L1				3				1
	L2		1		6		2		11
	L3				7				6
	Mixed		1 (L2/M2 right side; M2 left side)		2 (both L2/M2)		1 (L1/M1 right side; L1 left side)		5 (1 L3/M3 and 4 L2/M2)
	femoral		1 (bilateral)		1				
Operating time (min)		66		57		93		68	
Complication			0		1		0		1

*l-TAPP* laparoscopic TAPP, *r-TAPP* robotic TAPP, *EHS* European Hernia Society

### Surgical data

The mean operating time for unilateral or bilateral hernia repair in the l-TAPP group was 57 and 66 min, respectively (range unilateral 33–105 min, bilateral 37–104 min). In the r-TAPP group the mean operating time for unilateral or bilateral hernia repair was 68 and 93 min, respectively (range unilateral 43–116 min, bilateral 65–95 min). There were no unplanned over-night stays or readmissions during the first postoperative days. One postoperative hematoma was noted in each group at the first postoperative check-up appointment and was treated conservatively. There was no 30-day mortality or morbidity, nor was any of the 58 patients readmitted within the first 30 days after surgical therapy.

### Postoperative pain

**Fig. 1:**
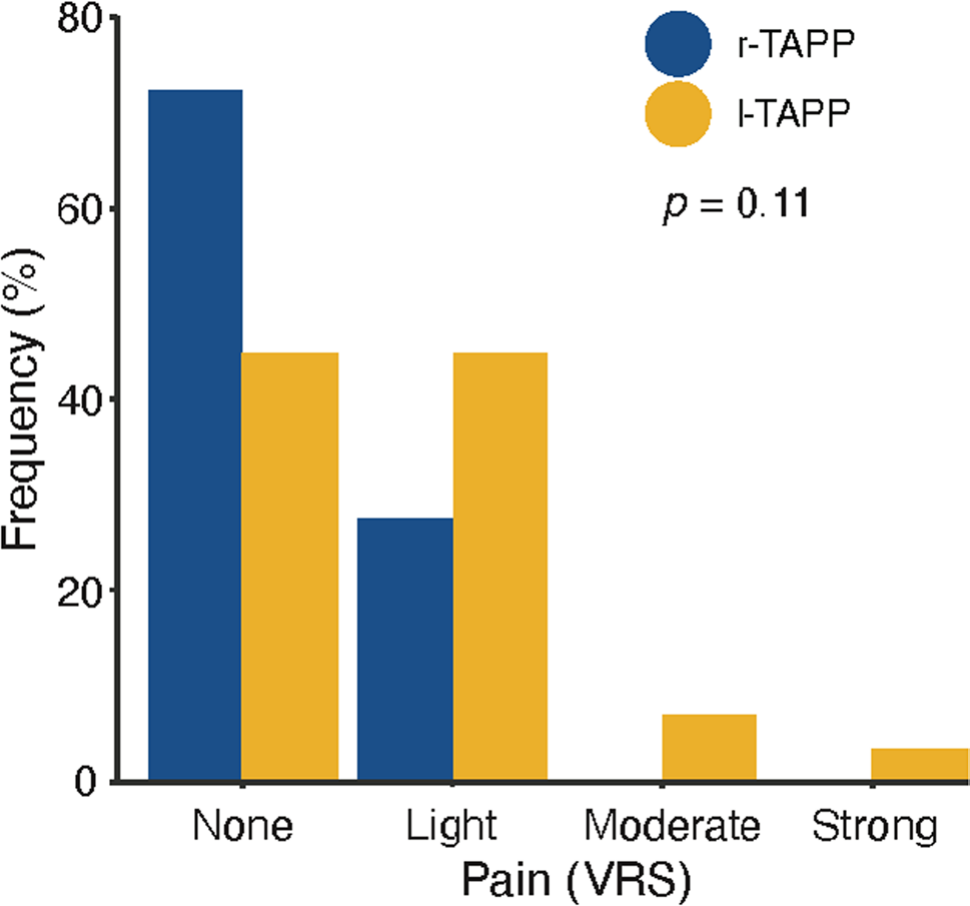
Assessment of postoperative pain level using the Verbal Rating Scale (VRS). Chi-squared contingency analysis did not demonstrate a statistical difference between r-TAPP (robotic TAPP) and l-TAPP laparoscopic TAPP (*p* = 0.11)

In the l-TAPP group 45% of the patients reported no postoperative pain and 45% had low postoperative pain. Moderate and/or severe postoperative pain was reported by 10% of patients following l-TAPP. The majority of the patients in the r-TAPP group (72%) were free of pain and 28% only reported a low level of pain. This trend towards lower pain level following r-TAPP did not reach statistical significance in the chi-squared contingency analysis (*p* = 0.11) (Fig. 1).

## Discussion

Our cohort study reports the short-term results of r-TAPP with the conventional l-TAPP in an outpatient setting. We found that r-TAPP can be performed in outpatient cases with a similar risk for perioperative complications within the first 30 postoperative days and reduced postoperative pain when compared to l-TAPP.

Minimal invasive, laparoendoscopic inguinal hernia surgery is superior compared to open inguinal hernia surgery regarding postoperative pain, wound healing, length of hospital stays, and time till return to work. Furthermore, a lower risk of chronic inguinal pain syndrome [1, 2, 4] and socioeconomic advantages have been reported for laparoendoscopic techniques [2].

The number of robotic operations in the field of hernia surgery is increasing [6]. In that context, r-TAPP has repeatedly been demonstrated to be a safe, efficient, and potentially superior alternative to l-TAPP in the treatment of inguinal hernias in an inpatient setting [7–9]. Accordingly, Ramser et al. recently reported unicentric results of 225 patients treated with r-TAPP, of which 80 patients were treated as outpatients [10]. In addition, r-TAPP may be superior to l-TAPP in case of complex or revisional inguinal hernia [9, 11].

Worldwide, there is a clear trend towards outpatient inguinal hernia surgery primarily to reduce health care costs. However, data comparing the outcomes of outpatient vs. inpatient inguinal hernia repair are limited but suggest that inguinal hernia surgery can be safely performed in an outpatient setting [12].

In our study, we found the mean operating time for r-TAPP to be 11 min longer for unilateral hernia repair compared to l-TAPP which was mainly due to the time necessary for the docking maneuver of the robotic system. Even though we did not explicitly record the respective time of the docking maneuver in our study, it seems possible to reduce this additional time with increased training [5, 13].

Operating time is an important factor with regard to the cost-effectiveness of r-TAPP. High acquisition and maintenance costs, as well as longer operating times, are the main drivers for the overall higher costs of r-TAPP compared to l-TAPP [14]. However, data are equivocal [15].

In line with previous data [15, 16] we found r-TAPP to be associated with lower postoperative pain compared to l-TAPP. To objectify postoperative pain perception, we used the VRS which is a simple unidimensional, validated test rating sensory components of pain. The VRS is considered to be superior to other unidimensional pain perception tests like the VAS (Visual Analogue Scale) in evaluating postoperative pain [17].

Our study has several limitations. First, this is comparative cohort study. Considering the very low complication rate of l-TAPP, a much larger number of patients would have been needed in a randomized controlled study design to allow a statement on the equivalence or even superiority of the r-TAPP compared to l-TAPP. However, as the robotic approach represents a refined version of laparoscopy, our study adds relevant information to the available literature and suggests that the r-TAPP may represent an alternative to l-TAPP in an outpatient setting with comparable safety.

Second, due to the standards of the health department of the canton of Zurich regulating the reimbursement for in- and outpatient treatment, only low risk patients were included in our analysis questioning the generalizability of our observations for high-risk patients.

Finally, the sample size is small, and the follow-up period is short. Therefore, we cannot make any statement about the medium and long-term results of outpatient robotic hernia surgery.

## Conclusion

We demonstrate that r-TAPP in an outpatient setting is associated with lower postoperative pain compared to outpatient l-TAPP in our institution. The future of r-TAPP as an option for outpatient hernia surgery is unlikely to be decided by perioperative quality parameters, which are largely equal, but primarily by the assessment of different treatment costs.
